# Supplementary material for: Carbohydrate Ingestion before Exercise for Individuals with McArdle Disease: Survey Evidence of Implementation and Perception in Real-World Settings
Source: Nutrients. 2024 May 9;16(10):1423. doi: 10.3390/nu16101423 (PMC11124166; doi:10.3390/nu16101423)
Supplement: Supplementary file 1 [file nutrients-16-01423-s001.zip › nutrients-2912968-supplementary.pdf]

Supplementary File S1.

Survey - The Effectiveness of Current McArdle Disease Management Guidelines of Nutrition and Exercise

The data collected will be used to guide further research into McArdle disease, so it is important to be as specific and precise as possible with your responses.

**Questions used for analysis in article*

I confirm that I am 18 years of age or older and have been diagnosed via DNA/GENETIC analysis to CONFIRM I have McArdle disease. I have read the Participant Information Sheet and I agree to the above statements and I give my informed consent to participate in the survey.

○ Agree (1)

*Q3 What is your height? (in centimeters)

____

*Q4 What is your current weight? (in kilograms)

____

*Q5 What is your age?

____

*Q6 What country do you live in?

____

*Q7 What is your gender?

- ○ Male (1)
- ○ Female (2)
- ○ Non-binary (3)

Q8 From the responses below how do you feel McArdle disease impacts on day to day life

- ○ I have problems with almost every activity of daily living and exercise (1)
- ○ I have problems with some activities of daily living. (2)
- ○ I only have problems with more intense activity. (3)
- ○ I only have problems when I exercise (4)
- ○ I can do anything I want and not be affected by my McArdle disease (5)

Skip To: Q10 If Q8 = I can do anything I want and not be affected by my McArdle disease

---

Q9 What daily activities do you find most difficult to perform with McArdle disease? (i.e. walking up stairs, washing your hands, opening jars, grating cheese, etc. Please enter N/A if you do not have any problems with daily activities)

____

____

____

____

____

---

*Q10 How many days a week do you perform continuous physical activity? This includes activities like running or walking, walking the dog, playing sport, working in the garden, riding your bike or walking to work, etc. (Any activity that would cause you to increase your heart rate and cause sweating for a prolonged period. This does not include resistance/weight training)

- Never (1)
- Once a week (2)
- 2-3 times a week (3)
- More than 3 times a week (4)

Skip To: Q12 If Q10 = Never

*Q11 On average, how long are you physically active for in one of the sessions from the previous question?

- 15 minutes or less (1)
- 15-30 minutes (2)
- 30-60 minutes (3)
- More than 60 minutes (4)

Q12 Do you currently undertake any resistance training/weight training?

- Yes (1)
- No (2)

Q13 Do you currently undertake any high-intensity exercise? (excluding resistance/weight training). For example, exercise that is greater than 75% of your maximal heart rate like sports involving repeated sprints or repeated jumping.

- No (1)
- Yes (2)

Q14 What time of day do you complete most of your physical activity for EXERCISE purposes? i.e. walking, riding, running, swimming, etc.

- Morning (before breakfast) (1)
- Morning (after breakfast) (2)
- Middle of the day (3)
- Evening (4)
- I do not do physical activity for exercise purposes (5)

---

*Q15 Do you find the current McArdle disease management guidelines of consuming sugary drinks or foods before you are physically active relieves/minimises your McArdle symptoms?

- Yes (1)
- No (2)
- Sometimes (3)
- I have never tried this management technique (4)

Skip To: Q18 If Q15 = I have never tried this management technique

---

*Q16 With regards to the current McArdle disease management guidelines of consuming sugary drinks or foods before you are physically active, what sugary drinks or foods have you tried before exercise?

- ▢ Fruit or fruit juice (please specify amount consumed) (1) ____
- ▢ Candy/lollies/sweets (please specify amount consumed) (2) ____
- ▢ 37g sucrose (3)
- ▢ Soft drink i.e. Cola, Fanta, Sprite, etc. (please specify amount consumed) (4) ____
- ▢ Energy drink. i.e. Redbull, Monster, etc. (please specify amount consumed) (5) ____
- ▢ Sports drink. i.e. Gatorade, Powerade etc. (please specify amount consumed) (6) ____
- ▢ Other (please specify) (7) ____

Q17 With regards to the current McArdle disease management guidelines of consuming sugary drinks or foods before you are physically active, what sugary drinks or foods do you find most effective at relieving your McArdle symptoms during exercise?

- ☐ Fruit or fruit juice (1)
- ☐ Candy/lollies/sweets (2)
- ☐ 37g sucrose (3)
- ☐ Soft drink i.e. Cola, Fanta, Sprite, etc. (4)
- ☐ Energy drink. i.e. Redbull, Monster, etc. (5)
- ☐ Sports drink. i.e. Gatorade, Powerade etc. (6)
- ☐ Other (please specify) (7) ____

Q18 During exercise have you ever become hypoglycemic (developed short-term low blood sugar)? Symptoms include dizziness, hunger, confusion, inability to concentrate.

- ○ Yes (1)
- ○ No (2)
- ○ I'm not sure (3)

Skip To: Q21 If Q18 = No

Q19 Please describe this hypoglycemic event in as much detail as possible. What activity were you doing when it occurred/occurs? What intensity was the activity you were undertaking? What time of day was it? Had you eaten within the last 2 hours before? How many years/weeks/days ago did it occur? etc.

____

____

____

____

____

Q20 How often do you develop hypoglycemia (low blood sugar) when exercising?

- Most of the time when I exercise (1)
- Roughly 50% of the time when I exercise (2)
- It has only happen a few times when I exercise (3)

Q21 Have you tried taking caffeine before being physical active to relieve your McArdle symptoms?

- Yes (1)
- No (2)

Skip To: Q24 If Q21 = No

Q22 Did you find caffeine reduced your McArdle symptoms (made you felt better)?

- Yes (1)
- No (2)
- I am unsure (3)

Q23 How did you consume the caffeine?

- ◯ Coffee or tea with sugar and milk (1)
- ◯ Coffee or tea with sugar but NOT milk (2)
- ◯ Coffee or tea with milk but NOT sugar (3)
- ◯ Coffee or tea with NO milk and NO sugar (4)
- ◯ Coffee or tea with sugar and cream (5)
- ◯ Coffee or tea with cream but NOT sugar (6)
- ◯ Energy drink (i.e. Redbull, Monster, V, etc.) (7)
- ◯ Caffeine tablet/powder (8)
- ◯ Soft drink (9)
- ◯ Diet soft drink (10)
- ◯ Other (11) ____

Q24 What statement best relates to you?

- ◯ My McArdle symptoms are reduced (you feel better) if I have breakfast in the morning (1)
- ◯ My McArdle symptoms are reduced (you feel better) if I DON'T have breakfast in the morning (2)
- ◯ I don't notice any difference if I do or don't have breakfast (3)
- ◯ I eat a particular breakfast in the morning that reduces (you feel better) my McArdle symptoms (please detail breakfast below) (4) ____

Q25 Where do you get the majority of your information about McArdle disease? (You can select multiple answers)

- ☐ Your general practitioner (GP) (1)
- ☐ IamGSD Facebook Group or website (2)
- ☐ Muscular Dystrophy websites (3)
- ☐ General internet health websites (4)
- ☐ YouTube (5)
- ☐ Scientific research papers (6)
- ☐ Magazines (7)
- ☐ AGSD Websites (8)
- ☐ Other (Please specify below) (9) ____

Q26 How many times have you been medically diagnosed with rhabdomyolysis (not self diagnosed, specifically diagnosed by a medical professional at a clinic or a hospital)?

- ○ Never (1)
- ○ Once (2)
- ○ 2-3 times (3)
- ○ More than 3 times (4)
- ○ I don't know what rhabdomyolysis is (5)

Skip To: Q29 If Q26 = Never

Q27 Did an episode of rhabdomyolysis diagnosed by a medical professional lead to the diagnosis of your McArdle disease?

- ◯ Yes (1)
- ◯ No (2)

Q28 How many times have you been diagnosed by a medical professional with acute renal failure due to your McArdle disease?

- ◯ I have never been diagnosed with acute renal failure (1)
- ◯ Once (2)
- ◯ 2-3 times (3)
- ◯ more than 3 times (4)
- ◯ I have been diagnosed with acute renal failure but it was not due to my McArdle disease (5)

Q29 What are the most effective techniques you use to reduce your McArdle symptoms?

____

____

____

____

____
